# Telehealth Home Support During COVID-19 Confinement for Community-Dwelling Older Adults With Mild Cognitive Impairment or Mild Dementia: Survey Study

**DOI:** 10.2196/19434

**Published:** 2020-05-22

**Authors:** Jessica Marian Goodman-Casanova, Elena Dura-Perez, Jose Guzman-Parra, Antonio Cuesta-Vargas, Fermin Mayoral-Cleries

**Affiliations:** 1 Department of Mental Health Regional University Hospital of Málaga Biomedical Research Institute of Malaga (IBIMA) Málaga Spain; 2 Department of Physiotherapy University of Málaga Biomedical Research Institute of Malaga (IBIMA) Málaga Spain

**Keywords:** telehealth, confinement, coronavirus, COVID-19, well-being, emergency response, public health, mental health, physical health, elderly, older adults, cognitive impairment, dementia

## Abstract

**Background:**

The public health emergency of coronavirus disease (COVID-19) is rapidly evolving worldwide; some countries, including Spain, have implemented restrictive measures. Populations that are vulnerable to this outbreak and its physical and mental health effects include community-dwelling older adults with mild cognitive impairment or mild dementia. Telehealth is a potential tool to deliver health care and decrease exposure risk.

**Objective:**

The aims of this study were to explore the impact of confinement on the health and well-being of community-dwelling older adults with mild cognitive impairment or mild dementia, to provide television-based and telephone-based health and social support, and to study the effects of a television-based assistive integrated technology, TV-AssistDem (TeleVision-based ASSistive Integrated Service to supporT European adults living with mild DEMentia or mild cognitive impairment).

**Methods:**

A telephone-based survey was administered in Spain to 93 participants in the TV-AssistDem clinical trial from March 25 to April 6, 2020.

**Results:**

Of the respondents, 60/93 (65%) were women. The mean age was 73.34 (SD 6.07), and 69/93 (74%) lived accompanied. Lockdown measures forced 17/93 respondents (18%) to change their living arrangements. Health status was found to be optimal in 89/93 respondents (96%), with no COVID-19 symptoms. Grocery and pharmacy outings were performed by family members of 68/93 participants (73%); 57 (61%) reported overall well-being, and 65 (70%) maintained their sleep quality. However, participants living alone reported greater negative feelings and more sleeping problems. Regarding leisure activities, 53/93 respondents (57%) took walks, 32 (35%) played memory games, 55 (60%) watched television, and 91 (98%) telephoned relatives. 58/93 (64%) respondents reported accessing moderate or too much COVID-19 information, 89 (97%) received it from television, and 56 (62%) stated that their understanding of the information was extreme. 39/93 (39%) respondents had contacted health and social services, while 29 (31%) requested information regarding these services during the telephone call. There were no significant differences in health and well-being between the intervention and control groups. Respondents with TV-AssistDem performed more memory exercises (24/93, 52% vs 8/93, 17.4%; *P*<.001) than control respondents.

**Conclusions:**

Our findings suggest that during COVID-19 confinement, the physical and mental health and well-being was optimal for the majority of our vulnerable population. However, those living alone reported greater negative psychological effects and sleeping problems. Measures adopted to address the negative experiences of confinement included keeping informed about the situation, accessing health and social services, having a support network that prevents risk of exposure to COVID-19 and guarantees food and medical supplies, a daily routine with maintained sleeping habits and leisure activities, staying physically and mentally active with cognitive stimulation exercises, and ensuring social connectedness using technology. Television sets were preferred technological devices to access COVID-19 information, watch television as a recreational activity, and perform memory exercises as an intellectual activity. Television-based telehealth support using TV-AssistDem demonstrated potential for cognitive stimulation.

**Trial Registration:**

ClinicalTrials.gov NCT03653234; https://clinicaltrials.gov/ct2/show/NCT03653234

## Introduction

Coronavirus disease (COVID-19) has been declared a global emergency by the World Health Organization (WHO); this disease has created a rapidly evolving situation which has forced the implementation of unprecedented restrictions to control its viral spread and mitigate its impact [1]. Spain has one of the highest burdens of COVID-19 worldwide, with 59.4% of cases reported in people aged 60 years and older [2]. In response to the outbreak, the Spanish government issued a royal decree (463/2020) to declare a 15-day national emergency, with the exceptional measure of a nationwide lockdown that started on March 15th and has been extended twice since [3]. This decree enforces social distancing, quarantine of people exposed to the disease, and home confinement of people who remain healthy, allowing only essential outings. This restriction of movement of daily life activities and separation from loved ones may be challenging and unpleasant. This experience may impact the physical and mental health and well-being of those who undergo it. Demographic factors, such as sex, age, and baseline health and well-being status, have been described as preconfinement predictors of greater impact [4].

Community-dwelling older adults are among the most vulnerable to the impact of this confinement. Their chronic conditions may be aggravated by the consequences of confinement [4]. Additionally, WHO underlines that people with mild cognitive impairment or mild dementia may face a greater challenge during the outbreak [5]. Their comprehension of the public health situation and the measures to follow, such as staying at home or wearing a mask, may be limited [6]. Confinement may lead to social isolation, which is a risk factor for health-related consequences [7] and increases risk of dementia and cognitive decline in older adults [8-10]. Moreover, facing a novel and unknown situation is a potential stressor, especially when cognition is compromised [4]. Furthermore, nonattendance of face-to-face activities, such as memory workshops and day care services, may worsen the cognition and functioning of this population [11]. Reduced support availability may consequently increase caregiver burden [6]. Alzheimer Europe recommends the following for people with mild cognitive impairment or mild dementia and their caregivers: building a support network; keeping well informed; guaranteeing food and medical supplies; enjoying leisure activities; staying physically and mentally active using Stimulus, among others, for cognitive stimulation; and keeping socially connected [12].

In the age of information and communications technology, technology home-based interventions [13] (smartphones, tablets, computers, smart televisions, virtual assistants, ambient assistive devices, etc.) can support most of the above recommendations; they facilitate information sharing and online shopping, provide access to sports and entertainment, increase social connectedness, etc. However, most importantly, these interventions enable the distribution of health-related information and services. Telehealth is a potential major tool to deliver routine health care and prevent the risk of viral exposure, especially for people at higher risk [14].

The burden of COVID-19 exerts pressure on health care services, social support services [3], and caregivers. Public health systems that had not proactively integrated telehealth are working reactively against the clock to respond to this urgent situation. Nationally, the Spanish government has launched a COVID-19 triage app, Asistencia COVID-19 [15]; regionally, routine care is being remotely managed via centralized telephone numbers (Salud Responde) [16]. These efforts are noteworthy; however, for years prior to this outbreak, Europe proactively invested in information and communications technology research in at-risk populations with programs such as the European Active and Assisted Living (AAL) Programme. TV-AssistDem (TeleVision-based ASSistive Integrated Service to supporT European adults living with mild DEMentia or mild cognitive impairment) is an AAL project that was selected for the Call for Proposals “Living well with dementia: The contribution of information and communications technology to integrated solutions for enabling the well-being of people living with dementia and their communities” [17].

TV-AssistDem is a European multicenter randomized controlled trial that evaluates a television-based assistive integrated service to support and improve the quality of life of people with mild cognitive impairment or mild dementia and provide relief to their caregivers. The components of TV-AssistDem are a digital set-top-box service based on Android technology with a television-based interface, a webcam, and a centralized back-end service with a web-based interface. TV-AssistDem facilitates remote support through data transmission and video interactivity between users, caregivers, and health care professionals.

The current study is nested in the TV-AssistDem project. To address the unexpected health emergency of COVID-19, the TV-AssistDem team rapidly adapted the service to provide tailored support. Detailed information on COVID-19 was offered through the functionality of health education with selected content from official sources, such as the WHO, the Spanish Ministry of Health, and local authorities. Videos on recommendations and basic care measures, such as hand washing, were uploaded. In addition to offering informational content, three of the established functionalities of TV-AssistDem guarantee physical and mental health and well-being, social connectedness, and cognitive stimulation. The Health Education functionality enables visualization of videos of physical activity at home; the Videocall functionality enables communication with loved ones and health professionals through videocalls; and the Memory Games functionality provides cognitive stimulation with Stimulus memory games [18].

The use of information and communications technology in reducing social isolation, improving cognition, and facilitating access to services in people with mild cognitive impairment or mild dementia has been broadly studied [13,19]. However, despite the growing global interest in telehealth during the COVID-19 pandemic [14,20], no study has yet explored the use of telehealth home support during COVID-19 confinement in people with mild cognitive impairment or mild dementia and the impact of confinement on this population.

The three aims of this study were to explore the impact of confinement on the physical and mental health and well-being of community-dwelling older adults with mild cognitive impairment or mild dementia, to provide television-based and telephone-based health and social support, and to study the effects of a television-based assistive integrated technology (TV-AssistDem). We hypothesized that people with access to TV-AssistDem would report greater physical and mental health and well-being.

## Methods

### Ethical Declarations

The current study is nested in the clinical trial TV-AssistDem (ClinicalTrials.gov NCT03653234) approved by the Malaga Province Research Ethics Committee (Comité de Ética de la Investigación Provincial de Málaga), approval number 1770-N-17. The substantial amendments derived from this new study were reviewed by the ethics committee and were granted a favorable opinion.

People with mild cognitive impairment or mild dementia and their caregivers provided written consent before taking part in the TV-AssistDem clinical trial as per protocol [18]. Taking into consideration the rights of the participants and to perform this study during this exceptional situation of confinement, we informed people with mild cognitive impairment or mild dementia and their caregivers at the beginning of the telephone interview that the reason for the call was additional follow-up within the framework of the project.

### Survey Development

Telephone-based survey research was conducted according to Gordon’s Functional Health Patterns [21] (Multimedia Appendix 1). Overall, quantitative strategies (questions with numerically rated items) were used for data collection of the health perception-health management and sleep-rest patterns. Qualitative strategies (open-ended questions) were used for the coping-stress tolerance, activity-exercise, and role-relationship patterns. Data were organized into previously coded and listed categories using a directed content analysis approach.

### Participant Identification and Recruitment

Researchers from the Biomedical Research Institute of Malaga contacted 100 community-dwelling older adults with mild cognitive impairment or mild dementia by telephone. Potential respondents were TV-AssistDem study participants from both the intervention and control groups who had met eligibility criteria for the TV-AssistDem trial and who had not dropped out of the study. Participants in the intervention group had been specifically trained in the use of TV-AssistDem and were given daily access to the service in their home environment. Participants in the control group received treatment as usual. Both groups received follow-up visits at 6 and 12 months. Inclusion criteria were age >60 years, self-perceived cognitive impairment or caregiver’s perception of cognitive impairment that was present for at least 6 months, score of 23-27 points on the Mini-Mental State Examination, independent living, informal caregiver, pharmacological treatment, and written consent. Exclusion criteria were a score >11 on the Geriatric Depression Scale, terminal illness, and specific conditions (cognitive, visual, motor, etc.) which could compromise the use of the system [18].

### Interview Process

Participants were contacted by telephone by health professionals (a mental health registered nurse clinical specialist and a neuropsychologist). The researchers had previously established relationships with the participants during the TV-AssistDem study. Potential respondents were considered unreachable when no answer was given to 3 different calls on 3 different days. The telephone call time frame was March 25 to April 6, 2020. The researchers interviewed the participants using the telephone-based survey. Caregivers were interviewed on behalf of people with mild cognitive impairment or mild dementia when cognitive or emotional statuses were compromised.

**Figure 1:**
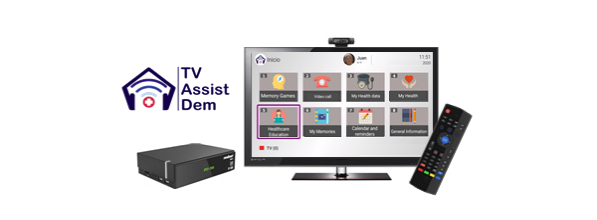
TV-AssistDem adapted to provide tailored support during COVID-19.

**Figure 2:**
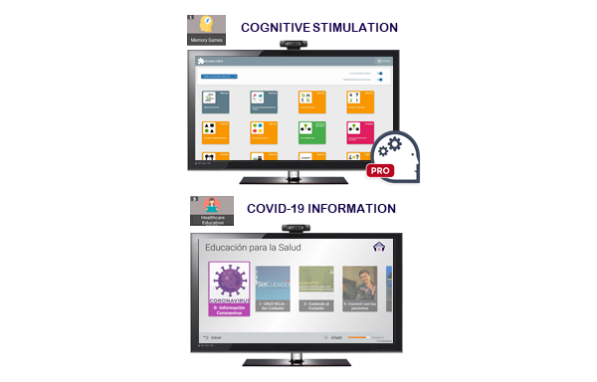
TV-AssistDem focused on cognitive stimulation and COVID-19 information.

**Figure 3:**
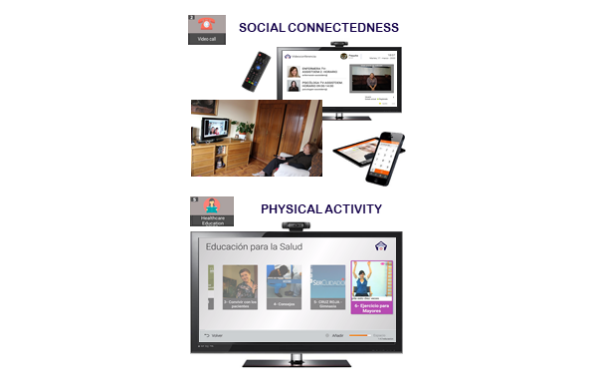
TV-AssistDem focused on social connectedness and physical activity.

During each interview, when necessary, health information and counselling was provided by the health professionals regarding COVID-19. This information included the symptoms and mode of transmission of the disease, contact telephone numbers for health care and social services to manage difficulties arising from the confinement situation, and recommendation guidelines for staying physically and mentally active. In addition, people with mild cognitive impairment or mild dementia in the intervention group were notified of the new updates available in the Health Education functionality TV-AssistDem concerning COVID-19. These included selected content from official sources such as the WHO, the Spanish Ministry of Health, and local authorities. Informative content included infographics and videos about the disease, basic protection measures such as hand washing, advice for managing psychological distress, and guidelines to carry out health procedures. Additionally, the participants were encouraged to continue to use three of the usual functionalities of TV-AssistDem which are meant to guarantee physical and mental health and well-being. Physical activity was promoted through the visualization of videos of indoor home exercise. Cognition was stimulated with Stimulus memory games. Lastly, social connectedness with loved ones and health professionals was facilitated through videocalls (Figures 1-3).

### Data Analysis

The chi-square test and Fisher exact test (when fewer than 80% of the expected frequencies of the cell were greater than 5) were used for analysis of the categorical variables. For quantitative variables, the Student *t* test was used. The analysis was performed by following an intention-to-treat procedure. The R program (version 3.6.2) was used for the analysis.

## Results

### Participants

A total of 93/100 (93.0%) TV-AssistDem study participants were successfully contacted: 47/93 (51%) in the intervention group and 46/93 (49%) in the control group. We could not reach 7/100 (7.0%) of the participants. The mean duration of the telephone calls was 12 minutes and 4 seconds (SD 7 seconds). For 21/93 (22%) participants, caregivers were interviewed on behalf of people with mild cognitive impairment or mild dementia whose cognitive or emotional statuses were compromised.

### Sociodemographics

The sample of people with mild cognitive impairment or mild dementia had a mean age of 73.34 years (SD 6.07); 60/93 (65%) were women, and 69 (74%) lived accompanied. Lockdown measures forced 17/93 participants (18%) to change their living arrangements (Table 1).

**Table 1:** Sample characteristics and differences between the intervention and control groups regarding demographic characteristics and living arrangements.

Characteristic		Total (N=93)	Intervention (n=47)	Control (n=46)	Statistical difference	*P* value
Age (years), mean (SD)		73.34 (6.07)	74.00 (6.16)	72.67 (5.98)	*t*_91_=1.053	.29
**Sex, n (%)**						
	Male	33 (36)	16 (34)	17 (37)	χ^2^_1_=0.09	.77
	Female	60 (65)	31 (66)	29 (33)		
**Change in living arrangements due to lockdown, n (%)**						
	Yes	17 (18)	10 (21)	7 (15)	χ^2^_1_=0.57	.45
	No	76 (82)	37 (79)	39 (85)		
**Living arrangements, n (%)**						
	Alone	24 (26)	14 (30)	10 (22)	χ^2^_4_=3.75	.44
	Spouse	39 (42)	22 (47)	17 (37)		
	Children	12 (13)	5 (11)	7 (15)		
	Spouse and children	13 (14)	4 (9)	9 (19)		
	Other	5 (6)	2 (4)	3 (7)		

### Functional Health Patterns

The health status of the participants was mainly found to be optimal; 89/93 (97%) presented no COVID-19 symptoms. Grocery and pharmacy outings were performed by family members for 68/93 participants (73%). Most of our respondents did not report inadequate or insufficient food supplies, nor did they report being unable to obtain regular medical care or prescriptions. 57/93 (61%) respondents reported overall well-being, and 65 (70%) maintained their quality of sleep (Table 2). Negative experiences reported included fear of becoming infected or infecting family members, frustration and boredom due to not being able to take part in daily activities, loss of usual routine, and social isolation. Leisure activities included physical, intellectual, recreational, and social activities. Of the 93 participants, 53 (57%) took walks, 32 (35%) played memory games, 55 (59%) watched TV, and 91 (98%) telephoned their family and friends (Table 3). Off-protocol, numerous respondents mentioned during the interviews that religious activities such as listening to religious programs on the radio felt comforting, and joining their neighbors at 8 PM for the national clapping against COVID-19 felt uplifting and provided a powerful reminder that although restrictions are followed in isolation, we are all part of a community.

**Table 2:** Differences between the intervention and control groups regarding health status and management during the COVID-19 pandemic.

Health status		Total (N=93), n (%)		Intervention (n=47), n (%)		Control (n=46), n (%)	Chi-square (*df*)		*P* value	
**Health status (COVID-19)^a^**										
	No symptoms		89 (96)		45 (96)	44 (96)		1.33 (2)		.51
	Symptoms without test		3 (3)		1 (2)	2 (4)				
	Symptoms and positive test		0 (0)		0 (0)	0 (0)				
	Hospitalized		0 (0)		0 (0)	0 (0)				
	ICU^b^ inpatient		1 (1)		1 (2)	0				
	Deceased		0		0	0				
**Health management: groceries/pharmacy**										
	Patient		12 (13)		3 (6)	9 (20)		9.19 (5)		.10
	Patient and family member		7 (8)		3 (6)	4 (9)				
	Family member		68 (73)		35 (75)	33 (72)				
	Home worker		1 (1)		1 (2)	0 (0)				
	Online		1 (1)		1 (2)	0 (0)				
	Other		4 (4)		4 (9)	0 (0)				
**Mental health and well-being**										
	Well		57(61)		27 (59)	30 (65)		0.41 (1)		.52
	Calm		8 (9)		3 (7)	5 (11)		0.55 (1)		.46
	Sad		27 (29)		17 (37)	10 (22)		2.57 (1)		.11
	Worried		20 (22)		7 (15)	13 (28)		2.30 (1)		.13
	Afraid		10 (11)		6 (13)	4 (9)		0.450 (1)		.50
	Anxious		22 (24)		8 (17)	14 (30)		2.15 (1)		.14
	Bored		13 (14)		6 (13)	7 (15)		0.09 (1)		.74
**Sleep quality**										
	Maintained		65 (70)		35 (81)	30 (68)		2.01 (1)		.16
	Altered		22 (24)		8 (19)	14 (32)				
	Unknown		6 (6)		0 (0)	0 (0)				

^a^COVID-19: coronavirus disease.

^b^ICU: intensive care unit.

**Table 3:** Differences between the intervention and control groups regarding activities.

Activity category and type		Total (N=93), n (%)	Intervention (n=47), n (%)	Control (n=46), n (%)	Chi-square (*df*)	*P* value
**Physical**						
	None	12 (13)	8 (19)	4 (9)	1.43 (1)	.23
	Walking	53 (57)	23 (49)	30 (65)	2.51 (1)	.11
	Stair climbing	10 (11)	5 (11)	5 (11)	0.01 (1)	.97
	Gymnastics	19 (20)	12 (26)	7 (12)	1.52 (1)	.22
	House chores	8 (9)	6 (13)	2 (4)	2.09 (1)	.27
	Other	12 (13)	3 (6)	9 (20)	3.59 (1)	.058
**Intellectual**						
	Memory exercises	32 (35)	24 (52)	8 (17)	12.22 (1)	<.001
	Reading	24 (26)	13 (28)	11 (24)	0.22 (1)	.63
	Playing games	6 (7)	1 (2)	5 (11)	2.85 (1)	.20
	Needlework	18 (20)	6 (13)	12 (26)	2.49 (1)	.11
	Painting	8 (9)	5 (11)	3 (7)	0.55 (1)	.71
**Recreational**						
	Watching television	55 (60)	28 (61)	27 (59)	0.04 (1)	.83
	Listening to radio or music	9 (9.8)	3 (6.5)	6 (13)	1.11 (1)	.48
	Playing with information and communications technology	8 (8.7)	4 (8.7)	4 (9)	0.00 (1)	>.99
	House chores	41 (45)	18 (39)	23 (50)	1.10 (1)	.29
	Keeping pets or plants	12 (13)	2 (4)	10 (22)	6.13 (1)	.01
**Social**						
	Home visits	46 (50)	24 (51)	22 (48)	0.10 (1)	.75
	Calls	91 (98)	46 (98)	45 (98)	<0.001 (1)	.99
	Videocalls	45 (48)	23 (49)	22 (48)	0.01 (1)	.91
	Texting	46 (50)	25 (53)	21 (46)	0.53 (1)	.47

### Knowledge of COVID-19 Situation and Health and Social Services

Of the 93 respondents, 58 (64%) reported accessing moderate or too much COVID-19 information, 89 (96.7%) learned about COVID-19 from television, and 56 (62%) described their understanding of the information as extreme. Moreover, 39/93 (38%) respondents had contacted health and social services, while 29 (31%) requested information regarding these services during the telephone call (Table 4).

**Table 4:** Differences between the intervention and control groups regarding information and resources pertaining to the COVID-19 pandemic.

Characteristic		Total (N=93), n (%)	Intervention (n=47), n (%)	Control (n=46), n (%)	Chi-square (*df*)	*P* value
**Amount of COVID-19^a^** **information accessed**						
	None	0 (0)	0 (0)	0 (0)	2.55 (3)	.47
	Too little	9 (10)	3 (7)	6 (13)		
	Moderate	29 (32)	15 (33)	14 (30)		
	Too much	29 (32)	17 (38)	12 (26)		
	Extreme	24 (26)	10 (22)	14 (30)		
**COVID-19 information source**						
	Family and friends	47 (51)	27 (59)	20 (43)	2.13 (1)	.14
	Television	89 (97)	45 (98)	44 (96)	0.34 (1)	.56
	Newspaper	5 (5)	3 (7)	2 (4)	0.21 (1)	.65
	Digital media	11 (12)	5 (11)	6 (13)	0.10 (1)	.75
	Radio	11 (12)	6 (13)	5 (11)	0.10 (1)	.75
**Understanding of COVID-19 information**						
	None	0 (0)	0 (0)	0 (0)	2.85	.41
	Too little	7 (8)	2 (4)	5 (11)		
	Moderate	13 (14)	8 (18)	5 (11)		
	Too much	15 (17)	9 (20)	6 (13)		
	Extreme	56 (62)	26 (58)	30 (65)		
**Resources contacted**						
	None	54 (61)	30 (68)	24 (55)	3.17 (3)	.37
	Health services	32 (36)	14 (32)	18 (41)		
	COVID-19 services	1 (1)	0 (0)	1 (2)		
	Emergency services	0 (0)	0 (0)	0 (0)		
	Social services nongovernmental organization	1 (1)	0 (0)	1 (2)		
**Resources used**						
	Health services number	12 (13)	3 (6)	9 (20)	3.59 (1)	.058
	COVID-19 services number	1 (1)	0 (0)	1 (2)	-	-
	Social services nongovernmental organization number	3 (3)	1 (2)	2 (4)	0.62 (1)	.49
	TV-AssistDem Health Education	13 (14)	13 (14)	N/A^b^	N/A	N/A

^a^COVID-19: coronavirus disease.

^b^Not applicable.

### Differences Between Living Alone and With Others

In comparison with the participants living with others (69/93, 74%), the participants living alone (24/93, 26%) reported less well-being (35% vs 71%; χ^2^=9.61; *P*=.002), more anxiety (59% vs 41%; χ^2^=3.90; *P*=.048) and more sleeping problems (48% vs 19%; χ^2^=4.71; *P*=.03). They more frequently reported being sad (44% vs 25%; χ^2^=2.953; *P*=.09) and bored (26% vs 10%; χ^2^=3.613; *P*=.057); however, these last results were only marginally significant.

### Differences Between the Intervention and Control Groups

There were no significant differences between the intervention and control groups in any sociodemographic variables, health status variables, or other variables associated with COVID-19 (Tables 1-4). Similarly, there were no differences regarding health management, mental health, well-being, or sleeping problems. Respondents with TV-AssistDem performed more memory exercises than control participants (24/93, 52% vs 8/93, 17%; *P*<.001).

## Discussion

### Principal Results

Our findings show that at the time of assessment, the physical and mental health and well-being of our study participants with mild cognitive impairment or mild dementia was overall optimal, although living alone was found to be a risk factor for greater psychological negative impact and sleeping problems. Television-based health and social support were provided in the intervention group, and telephone-based support was provided when requested. Television sets stood out as the preferred technological devices to access COVID-19 information, watch television as a recreational activity, and perform memory exercises as an intellectual activity.

Our sample presented characteristics which have been described as preconfinement predictors for greater health and well-being impact: female sex, old age, and mild cognitive impairment or mild dementia [4,5]. However, the change in living arrangements suggests that some households decided to rearrange their support network for the duration of the confinement. Having a support group at home has been described as helpful during disease outbreaks [4]. Our findings are encouraging in that they demonstrate that guaranteeing basic supplies, performing meaningful activities, and ensuring understanding of the situation by providing information from available resources improve the experience of confinement, as described by Brooks et al [4].

Overall, the respondents experienced optimal health status at the time of data collection, which can be explained by the reduced risk of exposure due to decreased daily life outings; they also reported having adequate supplies, which has been reported to mitigate the consequences of quarantine [4]. Our findings regarding mental health and well-being 2 weeks into confinement coincide with those described in the literature for quarantines under 10 days. Our respondents expressed fear, frustration, and boredom, which are frequently expressed negative feelings during confinement [4].

Participation in meaningful activities goes beyond pleasure or entertainment in people with mild cognitive impairment or mild dementia and has shown benefits for cognition and functioning improving independence in instrumental activities of daily living [22]. Physical activity plays a role in enhancing and maintaining cognition [23]; while the outdoor daily life and physical activities of our respondents were restricted, the vast majority engaged daily in physical activities. Leisure activities involving intellectually high cognitive effort or social interaction have been associated with better cognition [24]. Considering that nonattendance of memory workshops and day care services and social isolation may worsen the cognition and functioning of this population, participating in memory games analogically or technologically using smartphones, tablets, computers, or television-based devices may slow the negative consequences of confinement on cognition. The fact that respondents with TV-AssistDem performed significantly more memory exercises than control respondents suggests the potential of television-based activity for cognitive stimulation. Recreational activities have also demonstrated benefits in dealing with challenging situations [24]; however, careful attention must be given to watching television, which is associated with a dose-response cognitive decline [25].

Evidence suggests that access to devices as smartphones, tablets, computers, and television-based devices, which facilitate connectedness and communication, may reduce feelings of isolation [4]; the sample in this study has access to these devices. Since social isolation and loneliness have been associated with poorer cognition [26], special attention must be drawn to people with mild cognitive impairment or mild dementia regarding social activities during confinement. Furthermore, setting up a specific telephone support line has been described as effective in terms of providing health and social support [4,27]. In addition, TV-AssistDem offers and will continue to offer videocall services during confinement to participants in the intervention group.

Inadequate information is generally a potential stressor when facing novel and unknown situations [4]. While poor information may prevent people from comprehending the severity of the situation and complying with the measures, being extremely informed may significantly impact their perception of the situation and cause extreme worry. People with mild cognitive impairment or mild dementia face additional risk, as their cognition and understanding of the information may be compromised. Considering our population characteristics and that their access to COVID-19 information was reported to be moderate to too much, their extreme understanding is exceptional. While television sets stand out as the main source of information, family and friends are the second most common source, which may explain the participants’ remarkable understanding of the situation.

To our knowledge, this is the first study to explore the impact of COVID-19 confinement on the physical and mental health and well-being of community-dwelling older adults with mild cognitive impairment or mild dementia and the use of telehealth home support during COVID-19 confinement; no systematic reviews or clinical trials have been registered regarding this study population and technology to date.

### Limitations

Although telephone call interviewing was the safest means to communicate with people with mild cognitive impairment or mild dementia during the COVID-19 pandemic, there were several drawbacks. The amount of information gathered and provided in a single telephone call is limited, and researchers were required to balance the time spent on each call. Furthermore, overloading people with mild cognitive impairment or mild dementia with a long interview is not advisable, as it may feel tedious and time-consuming; also, excessive information should not be provided, as they may not be able to understand or remember it all in one telephone call. To ensure telephone call standardization, researchers followed an exhaustive structured protocol.

The impacts on physical and mental health and well-being were assessed 2 weeks into confinement. Studies show that aggravation of physical chronic conditions and poorer mental health and well-being, specifically significantly higher posttraumatic stress symptoms, appear when a quarantine lasts longer than 10 days. As the duration of the Spanish confinement is not yet clear, the long-term impact and consequences will need to be assessed over time [4]. At the time of submission of this publication, the government had announced an extension of the period of confinement; therefore, new evaluations will be carried out to determine the effects of the prolonged duration of the confinement.

### Conclusions

TV-AssistDem, our television-based assistive integrated technology, has been demonstrated to go beyond its initial objective of telehealth home support, promoting active aging of the elderly in their own homes and reducing caregiver burden. It has emerged as a promising cognitive stimulation and telehealth tool to deliver health care and facilitate remote caregiver support during exceptional circumstances, such as the current COVID-19 outbreak.

Our findings suggest that living alone is a risk factor for mental health and well-being and sleep during the coronavirus disease confinement in people with mild cognitive impairment or mild dementia. Recommended measures to address the negative experience of confinement include keeping well informed about the situation and accessing health and social services, having a reliable support network that decreases risk of exposure to COVID-19 and guarantees food and medical supplies, establishing a daily routine with maintained sleeping habits and leisure activities, staying physically and mentally active with cognitive stimulation exercises, and ensuring social connectedness using technology. Our findings show the potential of television sets for informative, recreational, and intellectual purposes in this population.

The findings of this study are valuable and meaningful and contribute to the growing evidence of COVID-19 research across populations, with special attention to people with mild cognitive impairment or mild dementia and technologies. Research regarding the assessment of needs of people with mild cognitive impairment or mild dementia and their caregivers during the COVID-19 pandemic, as well as technology-based support interventions, are urgently globally needed [6,28]. Our approach to explore the uniqueness of this experience in vulnerable populations is appropriate and replicable. Telephone-based interventions during the COVID-19 pandemic to survey and raise health awareness in older adults have proven to be feasible when face-to-face measures are not possible during outbreaks [27].

Government preparedness and responses to situations of the magnitude of COVID-19 determine related outcomes and consequences that go beyond the disease itself and have political, economic, and social impacts. In the short term, this study aims to prepare countries that have yet to face similar governmental restrictions to plan accordingly to guarantee and protect the physical and mental health and well-being of their community-dwelling older adults with mild cognitive impairment or mild dementia. In the long term, this study will contribute to the preparedness for another possible future outbreak. The consequences of this outbreak may affect not only people in confinement and their caregivers but also the health care system, which will need to provide care to address these consequences. The need for remote approaches to outreach and screen people at risk of social isolation may stimulate enhanced implementation of telehealth information and communications technology, such as TV-AssistDem, in the care and support of vulnerable populations who suffer the effects of the COVID-19 pandemic.

## Appendix

Multimedia Appendix 1 Survey questions.

## Abbreviations

AAL: active and assisted living
COVID-19: coronavirus disease
ICU: intensive care unit
TV-AssistDem: TeleVision-based ASSistive Integrated Service to supporT European adults living with mild DEMentia or mild cognitive impairment
WHO: World Health Organization
