# Supplementary material for: Telehealth Home Support During COVID-19 Confinement for Community-Dwelling Older Adults With Mild Cognitive Impairment or Mild Dementia: Survey Study
Source: J Med Internet Res. 2020 May 22;22(5):e19434. doi: 10.2196/19434 (PMC7247465; doi:10.2196/19434)
Supplement: Multimedia Appendix 1 [file jmir_v22i5e19434_app1.docx]

# Survey questions regarding the experience of coronavirus disease confinement in community-dwelling older adults with mild cognitive impairment or mild dementia

# INFORMED CONSENT

Dear Mr./Mrs. “name of PMCI/MD”, I am “research name” of the TV-AssistDeM study in which you are participating. We would like to carry out an additional follow-up within the framework of this project, to study the effects of home confinement on your health and well-being. Participation is completely voluntary, and we will comply with the same ethical-legal considerations as the current study. During a half-hour interview we will ask you questions regarding your experience during confinement due to COVID-19. By agreeing to participate, you will help us better understand how community-dwelling older adults with memory problems handle home confinement. Understanding this will help services develop effective preventive programs and treatments that help people manage similar situations. It may be unpleasant to report these experiences, if you think that completing this interview may affect you in any way, we encourage you not to participate. Thank you for your cooperation.

# RESPONDENT

Person with mild cognitive impairment or mild dementia

Caregivers on behalf of person with mild cognitive impairment or mild dementia whose cognitive or emotional status is compromised

# GENERAL INFORMATION

- 1. Have your living arrangements changed due to confinement?

1. Yes
2. No
   1. Regarding your living arrangements, who are you currently living with?
3. Alone
4. Spouse
5. Children
6. Spouse and children
7. Other

# HEALTH PERCEPTION-HEALTH MANAGEMENT PATTERN

- 1. Regarding your health status and COVID-19 which of the following applies:

1. I have/have had no symptoms
2. I have/have had symptoms compatible with COVID-19 but have not had a test done
3. I have/have had symptoms compatible with COVID-19 and have had a test done
4. I am/have been hospitalized because of COVID-19
5. I am/have been in an ICU because of COVID-19
   1. Regarding your health management, who is doing the groceries during confinement?

I am

A family member and myself

### A family member

### A home worker

### We are doing online shopping

### Other

## How would you consider your access to COVID-19 information?

1. None
2. Too little
3. Moderate
4. Too much
5. Extreme

## Which information source are you using to access COVID-19 information?

1. Family and friends
2. TV
3. Newspaper
4. Digital media
5. Radio

## How would you consider your understanding of the COVID-19 information accessed?

1. None

Too little

Moderate

Too much

Extreme

## Have you contacted any healthcare services to manage your health status, demand, change and/or cancel medical appointments, renew medical prescriptions, etc.?

1. Yes
2. No

## Do you need contact information regarding healthcare services to manage your health status, demand, change and/or cancel medical appointments, renew medical prescriptions, etc.?

1. Yes
2. No

## Have you contacted COVID-19 services to receive council regarding changes in your health status related to COVID-19?

1. Yes
2. No

## Do you need contact information regarding COVID-19 services to receive council regarding changes in your health status related to COVID-19?

1. Yes
2. No

## Have you contacted emergency services to receive assistance regarding changes in your health status related to COVID-19?

1. Yes
2. No

## Do you need information contact information regarding emergency services to receive assistance regarding changes in your health status related to COVID-19?

1. Yes
2. No

## Have you contacted any social support services to request support regarding food or medication provision?

1. Yes
2. No

## Do you need contact information regarding social support services to request support regarding food or medication provision?

1. Yes
2. No

# COPING-STRESS TOLERANCE PATTERN

## Regarding your mental health and well-being during confinement, how would you say you are feeling?

CATEGORIES: Well, Calm, Sad, Worried, Afraid, Anxious, Bored.

# SLEEP-REST PATTERN

## How would is your sleep during confinement when comparing it to before?

1. Maintained
2. Altered
3. Does not know/answer

# ACTIVITY-EXERCISE PATTERN

## Which physical activities are you doing during confinement?

CATEGORIES: None, Walks, Stair climbing, Gymnastics, House chores.

## Which intellectual activities are you doing during confinement?

CATEGORIES: Memory exercises, Reading, Playing games, Needlework, Painting

## Which recreational activities are you doing during confinement?

CATEGORIES: Watching TV, Listening to radio or music, Playing with ICTs, House chores, Keeping pets or plants

# ROLE-RELATIONSHIP PATTERN

## Which social activities are you doing during confinement?

CATEGORIES: Home visits, Calls, Videocalls, Texting
